# Work–Family Conflict and Job Outcomes for Construction Professionals: The Mediating Role of Affective Organizational Commitment

**DOI:** 10.3390/ijerph17041443

**Published:** 2020-02-24

**Authors:** Jiming Cao, Cong Liu, Guangdong Wu, Xianbo Zhao, Zhou Jiang

**Affiliations:** 1 School of Economics and Management, Tongji University, Shanghai 200092, China; caojm@tongji.edu.cn (J.C.); liucong9393@163.com (C.L.); 2 School of Public Affairs, Chongqing University, Chongqing 400044, China; 3 School of Engineering and Technology, Central Queensland University, Sydney, NSW 2000, Australia; b.zhao@cqu.edu.au; 4 College of Business, Government & Law, Flinders University, Adelaide 5042, Australia; zhou.jiang@flinders.edu.au

**Keywords:** work–family conflict, affective organizational commitment, job satisfaction, job performance, construction professionals

## Abstract

This study developed and tested a model, which involves the effects of work–family conflicts on job satisfaction and job performance of construction professionals, with a focus on the mediating role of affective organizational commitment. A structured questionnaire survey was conducted among construction professionals in China, resulting in 317 valid responses. The results, generated from structural equation modelling, revealed two interrelated dimensions of work-family conflicts, work’s interfering with family life and family life’s interfering with work. We found these two types of work-family conflicts directly, negatively affected affective organizational commitments and job satisfaction but not job performance. Additionally, affective organizational commitment positively affected job satisfaction and job performance, and mediated the effects of work–family conflicts on job satisfaction. This study advances our understanding of how or why work–family conflicts produce dysfunctional effects on employees’ job outcomes in the context of construction projects.

## 1. Introduction 

As a labor-intensive industry, the construction industry is characterized by high risks, heavy workloads, and long construction periods [1]. The demanding working environment in this industry involves long working hours [2,3], for construction projects are always of temporary, uncertain, and dynamic nature and tend to be in large, complex, and integrated scales [4]. These characteristics can lead to difficult tasks, complex processes, and unforeseen problems during the implementation of a project [5]. To achieve the ultimate goal of project success, many construction enterprises encourage workers to devote more energy and time (e.g., sacrificing evenings, weekends, and holidays) to work [6]. Thus, it is not surprising that researchers found construction professionals to work much longer hours, typically, at least six days a week, than contracted [7]. Construction professionals include the technicians, as well as the middle and senior managers, of the owners’ teams, contractors’ teams, supervisors’ teams, consultants’ teams, and designers’ teams [1]. Clearly, the heightened work demands have limited their abilities to fulfill family responsibilities [8]. Meanwhile, construction professionals are faced with demands from the family domain, such as raising children, accompanying spouses, and caring for elderly family members. Such family demands are also likely to affect their work [9]. Given the increasing number of dual-earner couples and single-parent workers, as well as the increasing responsibilities for elder care, work–family conflicts (WFC) have become a prominent issue in the construction field [10]. 

WFC is a form of inter-role conflict derived from the incompatibility between the role stressors from the work and family domains [11]. As a two-way concept, WFC includes work’s interfering with family responsibilities (WIF) and family responsibilities’ interfering with work (FIW). These WFC forms involve distinct features but both are associated with employees job-related outcomes [12]. For example, WIF, while primarily caused by excessive work demands, does harm to not only family well-being but also job satisfaction [13]. FIW, mainly determined by family demands, can distract employees from work, constrain their abilities to complete tasks, and ultimately affect their job performance [14]. In the field of construction project management, many studies on WFC have focused on the organizational-level or project-level outcomes such as project performance [5,7,8], having to some extent neglected the individual-level job outcomes. As a result, it is less clear how different types of WFC may drive construction professionals’ job outcomes, such as job satisfaction and job performance. 

To advance our knowledge in this field, we explore, in the construction project context, how WFC influence employees’ job outcomes by focusing on the associated mediation mechanism. We argue that WFC has negative effects on job satisfaction and job performance because it hinders construction professionals from affectively committing to the organization. Specifically, we consider employees’ affective organizational commitment (AOC), defined as “emotional attachment to, identification with, and participation in the organization” [15], as a mediator that transmits the effect of WFC to job outcomes. This consideration is first driven by the empirical hints that high WFC undermines AOC, in either organizations that mainly consist of permanent work groups or organizations that rely on temporary and dynamic construction project [16,17]. Research also suggests that AOC, as the fundamental factor determining employee dedication and loyalty [15,18], is an important predictor of individual outcomes that benefit the organization in general [13,19,20]. For example, previous studies in organizations characterized more by permanent tasks and groups have found that AOC increases employee wellbeing and performance and reduces absenteeism and turnover [21,22,23]. This line of work indicates the likelihood of WFC eliciting reduced AOC as well as the possibility of AOC driving employee outcomes (e.g., wellbeing and performance). Therefore, as we will discuss later, this study tests AOC as a mediator linking WFC to job satisfaction and job performance under the construction project setting. 

In brief, we draw on inter-role conflict theory and introduce AOC as a mediating variable to construct a theoretical model, which guides us to explore the relationships among WFC, AOC, and the job outcomes of Chinese professionals working on construction projects. Specifically, the objectives of this study are to test: (i) the influence of WFC on job satisfaction and job performance and (ii) the mediating role of AOC in the effects of WFC on these two job outcomes. By enriching and advancing the existing body of knowledge on WFC, this study contributes to an understanding of how AOC explains the influence of WFC on job outcomes in the context of construction projects. The study is also practically important, considering that it may serve as an effective reference for construction enterprises in terms of whether and how WFC can impact employees’ wellbeing and performance. 

## 2. Review of Major Concepts

### 2.1. Work–Family Conflict (WFC) 

WFC is a form of inter-role conflict in which the role pressures from the work and family domains are contradictory in some ways [15]. In the construction industry, WFC can be distinguished by work’s interfering with family responsibilities (WIF) and family responsibilities’ interfering with work (FIW) [12]. Construction professionals not only encounter long working hours and inflexible schedules but are also faced with family-related demands, such as raising children and caring for elderly family members [5,9]. According to inter-role conflict theory, the time and energy of an individual are limited [24]. However, work and family are competing for these limited resources [8]. Therefore, WIF and FIW are inevitable issues for construction professionals.

WIF includes time-based WIF, strain-based WIF, and behavior-based WIF [5,8,17]. Specifically, time-based WIF occurs when the time requirements from the work domain occupy the time of the family domain [25]. Because of the complexities, uncertainties, and high risks of construction projects [4], construction professionals must face uncertain tasks and complex processes that take time away from their family responsibilities [7]. Strain-based WIF occurs when the work stress limits their ability to meet the demands of the family domain [12]. During the implementations of construction projects, construction professionals need to tackle many stressful tasks, such as undertaking multiple roles and responding quickly to various emergencies [17]. The dynamic internal and external project environments, as well as stress-related tasks, are likely to trigger strain-based WIF [8]. Behavior-based WIF occurs when the behavior required in the work domain contradicts the behavior expected in the family domain [11]. For example, during uncertain and complex construction projects, construction professionals are likely to encounter difficulties and challenging tasks, which may lead to the display of negative emotions, such as disappointment and anger, and thus, the creation of poor reputations [26]. Therefore, construction professionals must maintain emotional resilience in the face of adversity and regulate their emotions appropriately to appear impersonal and dispassionate. However, family members prefer them to be passionate, emotional, and caring at all times [17]. The differences between the behavioral requirements of family and work are difficult to accommodate, and therefore, lead to behavior-based WIF. 

FIW includes time-based FIW, strain-based FIW, and behavior-based FIW [27]. Time-based FIW occurs when the time spent in the family domain reduces the time spent at work, thereby interfering with the performance of work duties [28]. Strain-based FIW is derived from stressful demands in the family domain that distract construction professionals from being able to fully engage with their work and complete tasks on time [5]. Behavior-based FIW arises when the behaviors of construction professionals expected in the family domain contradict those required in the work domain [11]. For example, solving family problems in a circumventive manner may not be suitable for solving problems at work [8]. 

### 2.2. Affective Organizational Commitment (AOC)

Organizational commitment includes behavioral commitment and psychological commitment [6]. Behavioral commitment involves the time and effort required by employees to achieve goals [29]. Psychological commitment refers to the willingness of employees to pursue goals and includes AOC, normative commitment, and continuance commitment [21,30]. AOC refers to an employee’s emotional attachment to and identification with their organization, whereas continuance commitment involves the perceived costs of leaving the organization [31]. Normative commitment results from perceived obligations toward the organization [18]. These three forms of commitment reflect the relationship between an employee and their organization, as well as affect their continued participation. Researchers generally believe that among the three forms of commitment, AOC has the greatest effects on the behaviors and willingness of employees [29,30,32].

AOC is a multi-dimensional concept. Morrow [33] regarded AOC as deriving from employees’ identification with the goals and values of their organizations, thereby motivating them to continue working in their organizations. Kim [19] indicated that AOC involved the degrees of employees’ identification with and attachment to their organizations’ cultures and goals. In general, AOC refers to employees’ emotional identification with, emotional participation in, and emotional attachment to their organizations [15]. Specifically, it includes three aspects: (i) identification with organizational values and goals; (ii) willingness to make efforts and changes for the organization; (iii) strong wish to remain a member of the organization [29]. Therefore, AOC is not only closely related to an employee’s emotions and willingness but also affects their attitudes and behaviors [30]. The AOC of construction professionals is derived from good organizational perceptions, which lead to positive emotions toward their organizations and good commitments to their projects [34]. Construction professionals with high levels of AOC can make more contributions than expected to their organizations because those who identify with and are emotionally attached to their organizations share the same goals with their organizations. They are also more willing to remain in their organizations and make more efforts to help achieve organizational goals, such as improving project performances and achieving smooth project deliveries [35]. Therefore, AOC is one of the key influential factors in the behaviors of construction professionals. High levels of AOC not only can motivate construction professionals to work harder to achieve organizational goals and make positive extra-role contributions but can also enhance their passion and enthusiasm for their organizations, thereby improving organizational cohesion, and ultimately, promoting the success of projects [36]. 

### 2.3. Job Outcomes

The job outcomes of construction professionals include job performance and job satisfaction [37]. The job performance of construction professionals refers to the results of tasks completed within their allotted times [30]. Job performance determines the career development and the promotion opportunities of construction professionals [38]. Not surprisingly, researchers have found that high performers tend to have stronger professional skills and better career opportunities, as well as tend to be more likely than low performers to be promoted within their organizations [39]. Construction projects are temporary projects involving multi-disciplinary professionals, so the job performances of construction professionals are affected by multiple interdependent processes and tasks [1,4]. In general, the job performance of construction professionals includes task performance, situational performance, and adaptive performance [40]. Task performance refers to the completion of project tasks and processes that involve task-related behaviors and activities [41]. Situational performance refers to the extensive psychological activities and social behaviors that occur during a project’s implementation [40,41]. Adaptive performance refers to the degree to which construction professionals adapt to changes in their work roles, as well as in the internal and external project environments [42]. Adaptions include creatively solving project problems, handling unpredictable project situations, and learning new techniques and project management regulations. 

The job satisfaction of construction professionals regards their emotional responses to work content, environments, and achievements [43]. In studies of project management, job satisfaction has always been a hot topic and has been studied as different types of variables. As an independent variable, job satisfaction has significant effects on many outcome variables, such as turnover intention, organizational commitment, and project performance [44,45,46]. As a dependent variable, job satisfaction has been proved to be affected by many factors, such as work environment, leadership style, and organizational culture [47,48,49]. As a mediating variable, job satisfaction plays a mediating role in many causal relationships. For example, Rezvani et al. [26] found that, in complex projects, job satisfaction mediates the relationship between emotional intelligence and project success. Huang and Su [50] pointed out that job satisfaction plays a mediating role between job training satisfaction and turnover intention. Additionally, previous studies have researched the moderating role of job satisfaction. Yang et al. [51] found that job satisfaction moderates the impact of leadership competency on project performance. The empirical research of Soomro et al. [52] indicated that job satisfaction moderates the relationship between work–life balance and employee performance. Therefore, job satisfaction has been proved to be associated with individual-related and organization-related outcomes in the field of construction projects. 

## 3. Theoretical Model and Hypothesis Development 

### 3.1. Theoretical Model

**Figure 1:**
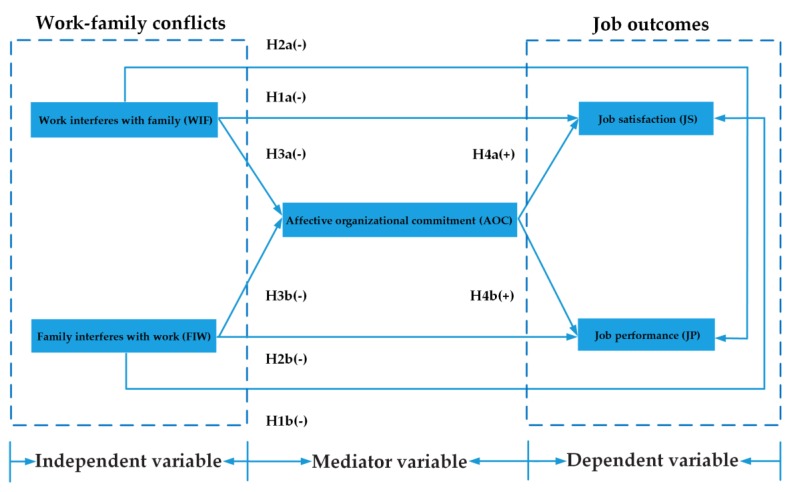
Theoretical model.

Researchers of project management consider WFC to be an antecedent variable and have explored the effects of WFC on the attitudes and behaviors, such as professional commitment, turnover intention, and project performance, of construction professionals [5,28,53]. However, how WFC affects job outcomes, namely, job satisfaction and job performance, through AOC remains unclear. To address this gap, our study applied inter-role conflict theory and adopted the input–mediator–outcome (IMO) model [54] to explore the relationships among WFC, AOC, job satisfaction, and job performance in the context of construction projects. In this study, the input variables were WIF and FIW, the mediating variable was AOC, and the output variables were job satisfaction and job performance. On the basis of the inter-role conflict theoretical perspective, a conceptual model was developed, as shown in Figure 1. 

### 3.2. Hypotheses Development

#### 3.2.1. The Relationship between WFC and Job Satisfaction 

Job satisfaction is an emotional state that reflects a construction professional’s internal satisfaction with their work environment, processes, and achievements [43]. Previous studies in permanent organizations have found a negative relationship between WFC and job satisfaction [31]. Construction projects are temporary projects that involve heavy workloads, complex tasks, and process arrangements, as well as dynamic internal and external settings [5]. The demanding work environment of a construction project has considerable potential to interfere with the family lives of construction professionals in a negative way [8]. WIF occurs when the work demands of construction professionals are contradictive with the fulfillment of their family responsibilities [7]. According to inter-role conflict theory, an individual has limited time and energy [24]. WIF reduces the time construction professionals must spend with their spouses and children or care for elderly family members, thereby leading to low levels of family well-being and job satisfaction [7]. FIW occurs when family responsibilities spill over into the work domain [55]. In many cases of FIW, construction professionals must deal with family issues during working hours [11]. However, doing so would consume their limited time and energy, even leading to their inability to complete tasks on time. The late completion of tasks could make them anxious, irritated, and even, angry [17]. These negative emotions would cause them to feel dissatisfied with both work and family, negatively affecting their attitudes and behaviors, such as professional commitment, job satisfaction, and job efficiency, at work [5,55]. With regard to job satisfaction, the following hypotheses are proposed:

**Hypothesis** **1a.** **(H1a).** *WIF negatively influences job satisfaction.*

**Hypothesis** **1b.** **(H1b).** *FIW negatively influences job satisfaction.*

#### 3.2.2. The Relationship between WFC and Job Performance

Job performance involves the completion of tasks within their allotted times [30]. Previous studies in permanent organizations have discussed the negative correlation between WFC and job performance [56,57]. A construction project is a temporary and dynamic project-based organization with the characteristics of long and irregular working hours, complex and challenging tasks, and changing project requirements; hence, construction professionals devote much energy and time to work [5]. According to inter-role conflict theory, the time and energy of an individual are limited [24], so the excessive occupation of time and energy by one role would inevitably affect the fulfillment of the responsibilities of another role, eventually leading to conflicts between the roles [11]. Therefore, the excessive occupation of time and energy by work limits the abilities of construction professionals to effectively fulfill family responsibilities, ultimately leading to WIF [8]. The family pressure brought about by WIF always leads to dissatisfaction with work and negative emotions, such as disappointment, frustration, and guilt, ultimately resulting in low levels of work efficiency and performance [17]. FIW is caused by issues in the family domain spreading to the work domain and always leads to distraction, as well as the loss of energy and time spent working, thereby leading to low levels of work efficiency and performance [55,58]. With regard to job performance, the following hypotheses are proposed:

**Hypothesis** **2a.** **(H2a).** *WIF negatively influences job performance.*

**Hypothesis** **2b.** **(H2b).** *FIW negatively influences job performance.*

#### 3.2.3. The Relationship between WFC and AOC

Inter-role conflicts occur when an individual’s role pressures from multiple domains are incompatible in some way or when they must play multiple roles [11]. Individuals can take measures to avoid inter-role conflicts. However, it is not possible for them to completely avoid inter-role conflicts, especially WFC, which arises from the work and family domains [59]. WFC reflects an imbalance between work roles and family roles, i.e., work can interfere with family life and vice versa [8]. Inter-role conflict theory states that the time and energy of an individual are limited, but different roles compete for these limited resources, and so, create conflicts among the roles [24]. For construction professionals, long working hours, heavy workloads, and inflexible schedules limit their abilities to perform their family responsibilities effectively [7]. Specifically, as technicians or managers, construction professionals must tackle many issues, such as the demands of internal and external stakeholders, complex tasks, and client requirements, that may increase or change. Such issues take up much personal time and do not allow the professionals to effectively fulfill their family duties, ultimately leading to WIF [17]. In many cases, the family pressures brought about by WIF make construction professionals feel dissatisfied with their work and lead to negative emotions, such as depression and guilt, which undermine their emotional identification and attachment to their organizations, ultimately leading to decreases in their levels of AOC [16]. Additionally, when family roles spill over into work roles, FIW is generated and interferes with work, creating stress and anxiety for the professional. Such situations may result in their inability to complete project tasks on time and the possibility of losing their job [17]. Therefore, FIW always leads to negative emotions, such as anxiety and frustration, which have negative effects on AOC [60]. Therefore, the following hypotheses were proposed:

**Hypothesis** **3a.** **(H3a).** *WIF negatively influences AOC.*

**Hypothesis** **3b.** **(H3b).** *FIW negatively influences AOC.*

#### 3.2.4. The Relationship between AOC and Job Outcomes 

AOC reflects the emotional attachment of employees who wish to stay in their organizations, whereas continuance commitment and normative commitment involve a cost or obligation mindset [35]. Therefore, employees with high levels of AOC want to participate in organizational actions because they identify with and are emotionally attached to the organizations [16]. AOC has been the focus of research in organizational behavior and has been proved to be closely related to individual-related behaviors and organization-related outcomes [19,33]. For example, Meyer et al. [21] found that AOC had the strongest and most favorable relations with organization-related outcomes, such as organizational performance and attendance. Through empirical research, Meyer and Herscovitch [61] found that AOC had stronger effects on employees’ behaviors and was related to a range of outcomes wider than achieved with continuance commitment and normative commitment. A construction project is a temporary system composed of multi-disciplinary professionals and a project team has the characteristics of diversity, multi-disciplinary knowledge, dynamics, and temporality [17]. The ultimate goal of a project team is to achieve project success by controlling project costs and quality, improving the economic, social, and environmental benefits of the project, and providing smooth project delivery within the specified period of time [4]. A construction professional with high levels of emotional attachment to and emotional identification with their organization is willing to contribute their efforts, as well as their professional knowledge and skills, to a project [30]. Team cohesion and passion for their organization would also be enhanced, thereby contributing to job efficiency, job satisfaction, and job performance [36]. With regard to these two factors, the following hypotheses are proposed:

**Hypothesis** **4a.** **(H4a).** *AOC positively influences job satisfaction.*

**Hypothesis** **4b.** **(H4b).** *AOC positively influences job performance.*

#### 3.2.5. The Mediating Role of AOC in the Effects of WFC on Job Outcomes 

Many scholars have found that conflicts between employees’ work roles and family roles have a critical impact on their job outcomes [31,56,57,62]. However, the potential impact mechanism of WFC on job outcomes is unclear, thus further research is needed. Considering the impact of conflicts on employees’ psychological factors [16,60,63] and the effect of psychological factors on their behaviors and attitudes [19,64,65], this study introduces AOC to explore the indirect impact of WFC on job outcomes. AOC is an emotional bond between employees and their organizations [35]. Employees with high levels of AOC are characterized by their attachment to the organization, their identification with the organization’s goals and values, a feeling of pride in their organization, as well as a strong desire to remain a member of the organization [32]. As a result, employees with strong AOC are more likely to adhere to the organization’s values and goals, even if these required behaviors extending beyond in-role responsibilities [66]. Specifically, employees with strong AOC have a strong sense of ownership and consider the interests of the organization as their own. When problems arise, instead of giving up, such employees are more likely to share ideas, give warnings, or encourage constructive changes [67,68]. 

WFC has become a serious problem in the construction industry, which brings great pressure to construction professionals [5]. Following the arguments of self-justification [69], employees who are stressed as a result of conflicts between their work roles and family roles tend to attribute such pressures to high-intensity work and family activities, become frustrated, and experience low levels of AOC [55]. Since AOC is a driving force of positive behaviors and attitudes of employees [70], reduced AOC will subsequently lead to low objective job performance and subjective job satisfaction of construction professionals [30]. Hypotheses 1a, 1b, 2a and 2b indicate that WFC has negative effects on job satisfaction and job performance. Hypotheses 3a and 3b suggest that WFC elicits reduced AOC. H4a, H4b and this section indicate that reduced AOC can undermine job satisfaction and job performance. These factors all combine to suggest a mediating role of AOC on the relationship between WFC and job outcomes. Therefore, the following hypotheses are proposed:

**Hypothesis** **5a.** **(H5a).** *AOC mediates the relationship between WIF and job satisfaction.*

**Hypothesis** **5b.** **(H5b).** *AOC mediates the relationship between FIW and job satisfaction.*

**Hypothesis** **6a.** **(H6a).** *AOC mediates the relationship between WIF and job performance.*

**Hypothesis** **6b.** **(H6b).** *AOC mediates the relationship between FIW and job performance.*

## 4. Variable Measurement and Pilot Test

### 4.1. Questionnaire Design

To test the conceptual model, a questionnaire was designed to measure the studied variables. Basic demographic data, such as family information and work status, were also investigated. Specifically, the measurements of the variables were composed of three parts: the two dimensions of WFC (independent variables), AOC (mediator variable), and the two dimensions of job outcome (dependent variables). The following three steps were used to modify and apply the measurement items of the variables. The first step was to identify and quote the measurement items that have been proved by prior studies to possess high-level reliability and validity [71]. Since the original scales were developed in English, all measurement items were back-translated and modified [72]. The second step was to improve the existing measurement items in combination with the practical situation of the Chinese construction industry [8]. The third step was to conduct on-site discussions with experts in the field of construction project management in order to optimize and confirm the measurement items [73]. 

The items used to measure WFC were designed according to previous studies (Boles et al. [74]; Netemeyer et al. [60]). The items used to measure AOC were designed with reference to the relevant literature (Meyer et al. [18]; Mowday et al. [29]). The items used to measure job outcomes were also designed according to previous studies (Babin and Boles [75]; Hartline and Ferrell [76]; Leung et al. [30]). Face-to-face interviews with experts were used to optimize the measurement items obtained from the literature and to ensure their applicability within the scope of construction projects [77]. Representatives of owners, contractors, supervisors, consultants, and designers were interviewed to collect their professional opinions on the appropriateness of the measurement items of WFC, AOC, and job outcome. We selected eleven experts from different project teams, who served as project managers, department managers, and project engineers. After two rounds of face-to-face discussions, we reached a consensus on the appropriateness of the measurement items (Table 1). All variable measurements in the questionnaire used a five-point Likert scale (from 1: ‘strongly disagree’ to 5: ‘strongly agree’). 

### 4.2. Pilot Test

**Figure 2:**
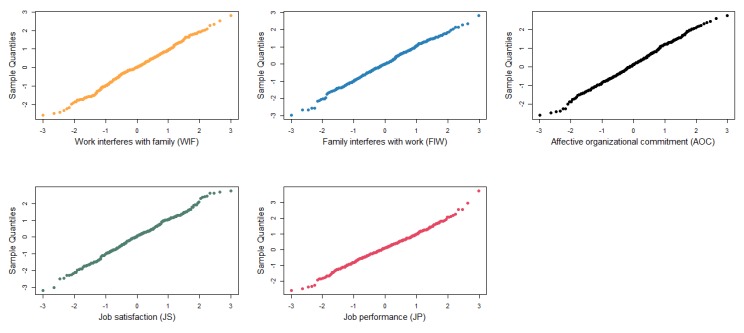
Q-Q plots of variables for the sample.

The purpose of the pilot test was to verify and modify the draft questionnaire [78]. The pilot test was conducted at construction projects of Shanghai, Zhejiang Province, Jiangsu Province and Jiangxi Province in China. Respondents included the technicians, as well as the middle and senior managers, of the owners’ teams, contractors’ teams, supervisors’ teams, consultants’ teams, and designers’ teams. A total of 374 questionnaires were sent out by email and express delivery. After testing the validity of 149 recovered questionnaires, 102 questionnaires were valid with an effective rate of 27% (102/374). Questionnaire screening identified and removed any questionnaire whose (1) answers were obviously not serious, (2) items were unanswered, (3) answers contradicted each other, and (4) answers were obviously similar in the same team [73]. Before the pilot test, we conducted a normality test on the valid sample. Specifically, we used the normal quantile–quantile plot (Q–Q plot), which is the most commonly used and effective diagnostic tool for testing if the data obeyed the normal distribution [79], which is shown in Figure 2. The sample distributions of WFC, AOC and job outcome, are almost linear. Therefore, the valid data did follow a normal distribution and could be further tested.

The pilot test consisted of three steps. First, the reliability coefficients of corrected-item total correlation (CITC) and Cronbach’s α were used to explore the reliability and validity of, as well as purify, all measuring items [4]. CITC reflected the reliability of the items. A CITC value below 0.5 meant that the item was to be deleted. Cronbach’s α was used to test internal consistency, which should not be below 0.7 [80]. Second, the Kaiser–Meyer–Olkin (KMO) test and the Bartlett test were used to assess if exploratory factor analysis could be implemented [81]. In this study, exploratory factor analysis was conducted for variables with KMO values greater than 0.6 [82]. The third step was to conduct an exploratory factor analysis. After the purification of the measurement scale, a formal questionnaire for large-scale sampling was formed.

### 4.3. Formal Data Collection

Since this survey had no sampling framework, a non-probability sampling method was adopted to obtain a representative sample [4]. This method was considered appropriate because the respondents were not selected randomly but according to their willingness to participate in the study [83]. The survey samples were collected from the technicians, as well as the middle and senior managers, of the owners’ teams, contractors’ teams, supervisors’ teams, consultants’ teams, and designers’ teams, of different construction projects in Shanghai, Jiangsu Province, Jiangxi Province, and Zhejiang Province. The survey construction projects include residential projects and public projects. The average construction period of these projects is 2−5 years, and the investment of these projects is about 500 million to 1.5 billion yuan (about US$72−214 million according to the exchange rate in February 2020). A total of 1100 questionnaires were distributed to the technicians, as well as the middle and senior managers, by email and express delivery. Of the questionnaires sent, 386 were returned. The criteria for questionnaire screening were the same as those for the pilot test. After screening, 317 of these questionnaires were valid with a response rate of 29% (317/1100), which was normal according to the norm of 20–30% in most construction project studies [84]. These valid data were used for reliability and validity analysis, as well as structural equation modeling (SEM) analysis. Before these analyses, we used the Q–Q plot to conduct a normality test of the data. The results showed that the data were consistent with a normal distribution. In addition, we analyzed the sample structure of valid questionnaires, whose categories and levels are shown in Table 2.

### 4.4. Confirmatory Factor Analysis

As part of SEM, the confirmatory factor analysis (CFA) was used to verify the reliability and validity of a measurement model. This analysis can confirm the suitability of the observed variables (measurement items) to each potential variable, as well as the overall reliability and internal consistency of the measurement items [4]. The CFA of WIF, FIW, AOC, job satisfaction, and job performance was conducted with AMOS 21.0. This analysis produced item reliability indicators and the factors of construct reliability (CR). Variable measurement items with standardized factor loads below 0.6 were deleted [85]. CR reflected the consistency among the measurement items. A CR value greater than 0.6 represented good construct reliability [86]. The average variance extracted (AVE) was used to test convergence validity. An AVE value greater than 0.5 indicated that the variable measurement items had good convergence validity [87]. Fitting indicators, such as the ratio of the chi-square statistic to the degrees of freedom ($x^{2}/\mathrm{df}$), root mean square error of approximation (RMSEA), goodness-of-fit index (GFI), comparative fit index (CFI), adjusted goodness-of-fit index (AGFI), incremental fit index (IFI), and the normed fit index (NFI), were used to assess the goodness-of-fit [86]. Specifically, $x^{2}/\mathrm{df}$ should be less than the strict limit of 3 [4]. A RMSEA value lower than 0.08 represented a good fit [86]. NFI, IFI, GFI, CFI, and AGFI should be higher than the threshold of 0.9 [4].

The CFA results were shown in Table 3. It can be seen that all the indicators of each research variable met the requirements and the standardized factor loads of all items exceeded 0.6. The CR value of each potential variable was greater than 0.7, indicating that the overall reliability and internal consistency of the measurement items were high. The AVE value of each potential variable was higher than 0.6, which showed good convergence validity. Additionally, the chi-square method and Harman’s single-factor test were applied to check non-response biases and the common method bias, respectively [88]. The results showed that the significant heterogeneity between the variables and the common method bias was not a serious problem in this study and signified that the theoretical model could be tested.

## 5. Model Testing and Results

### 5.1. Control Variables Test

SEM was used to test the theoretical model because it is considered a suitable tool for exploring the relationships between variables [89] and is widely used in studies of construction project management [4,86]. First, considering that the demographic variables, such as gender and marital status [40], may have an impact on the dependent variables, this study explored the impact of these control variables on job outcomes. The results show that gender and marital status have a non-significant effect on job outcomes (gender→job satisfaction, 0.063, *p* > 0.05; gender→job performance, −0.139, *p* > 0.05; marital status→job satisfaction, 0.015, *p* > 0.05; marital status→job performance, 0.029, *p* > 0.05). Second, considering that different job positions may affect job outcomes [90], this study examined the impact of this control variable on job outcomes. The results indicate that job positions had a non-significant impact on job outcomes (job position→job satisfaction, −0.012, *p* > 0.05; job position→job performance, −0.015, *p* > 0.05). Third, since older construction professionals may respond differently to WFC compared to young construction professionals [1], this study implemented a homogeneity of variance test (WIF (Levene Statistic = 0.801, *p* > 0.05); FIW (Levene Statistic = 0.367, *p* > 0.05)). The results show that the assumption of homogeneity of variance was effective, indicating that the response of elderly construction professionals to WFC is the same as that of young construction professionals.

### 5.2. Independent Samples t-Test

Considering that there are many more men than women in the large samples, it is necessary to test whether women differ from men in WIF, FIW, AOC and other study variables, so as to determine whether a grouping hypothesis test for male and female samples is required. The independent samples t-test of male group and female group was performed with SPSS 20.0. The results were shown in Table 4. As can be seen from Table 4, for WIF, there was no significant difference between female scores (Mean = 3.270, S.D. = 0.764) and male scores (Mean = 3.294, S.D. = 0.753, t = −1.721, *p* = 0.084 > 0.05). For FIW, there was no significant difference between female scores (Mean = 3.160, S.D. = 0.785) and male scores (Mean = 3.307, S.D. = 0.713, t = −1.641, *p* = 0.078 > 0.05). For AOC, there was no significant difference between female scores (Mean = 3.654, S.D. = 0.949) and male scores (Mean = 3.509, S.D. = 0.773, t = −1.493, *p* = 0.092 > 0.05). For job satisfaction, there was no significant difference between female scores (Mean = 3.479, S.D. = 0.926) and male scores (Mean = 3.721, S.D. = 0.629, t = −1.719, *p* = 0.067 > 0.05). For job performance, there was no significant difference between female scores (Mean = 3.137, S.D. = 0.758) and male scores (Mean = 3.209, S.D. = 0.853, t = −1.540, *p* = 0.075 > 0.05). Therefore, women do not differ from men in each potential variable, and subsequent SEM test do not require grouping of male and female samples.

### 5.3. SEM Test

**Figure 3:**
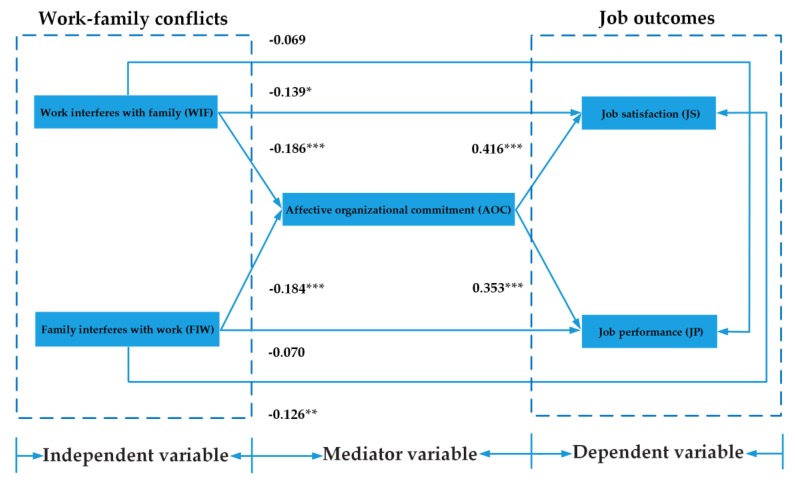
SEM test results of the theoretical model.

After testing the control variables, the analysis of the theoretical model was conducted with AMOS 21.0. The results are shown in Figure 3 and Table 5. It can be seen that the fit indicators meet the demands. Specifically, x^2^/df is 1.74, which is less than the strict limit of 3 [4]. RMSEA is 0.063, which is lower than 0.08 and is a good fit [86]. NFI, IFI, GFI, and AGFI are 0.96, 0.93, 0.94, and 0.92, respectively, all of which are higher than the threshold of 0.9 [91].

As can be seen from Table 5, most hypotheses have passed the test. First, the effects of WIF and FIW on job satisfaction are negative and significant (WIF→job satisfaction, −0.139, *p* < 0.05; FIW→job satisfaction, −0.126, *p* < 0.01), providing support for H1a and H1b, respectively. Second, the effects of WIF and FIW on job performance are not significant (WIF→job performance, −0.069, *p* > 0.05; FIW→job performance, −0.070, *p* > 0.05). Therefore, H2a and H2b are not supported. Third, the effects of WIF and FIW on AOC are negative and significant (WIF→AOC, −0.186, *p* < 0.001; FIW→AOC, −0.184, *p* < 0.001), providing support for H3a and H3b, respectively. Fourth, the effects of AOC on job satisfaction and job performance are positive and significant (AOC→job satisfaction, 0.416, *p* < 0.001; AOC→job performance, 0.353, *p* < 0.001), providing support for H4a and H4b, respectively.

### 5.4. Mediating Effect Test

This study needs to obtain the results of four mediating effect tests (i.e., a multiple mediating analysis). Although the mediating effect can be tested in AMOS through bootstrapping (n = 5000, 95% CI), AMOS can not directly provide a multiple mediating analysis. Specifically, for the multiple mediating analysis, AMOS cannot directly test the effects of each specific mediation, and can only produce a total mediating effect result. Therefore, we used the PROCESS mediation macro in SPSS to implement a multiple mediating analysis [92], as shown in Table 6. The bootstrap sample was set to 5000 and the statistical significance was evaluated using a 95% confidence interval (CI) [93,94]. As Hayes and Preacher [95] stated, PROCESS is a general computational tool for reliably evaluating multiple meditators in parallel that uses bootstrapping and Sobel’s Z-scores to generate boot confidence intervals (boot CI) and effect sizes of indirect effects. When boot 95% CI excludes 0, the indirect effect is statistically significant [93]. As outlined in Table 6, AOC mediating the relationship between WIF and job satisfaction and the relationship between FIW and job satisfaction is confirmed (−0.114, boot 95% CI= [−0.170, −0.068]; −0.115, boot 95% CI= [−0.172, −0.069]), providing support for H5a and H5b, respectively. Additionally, AOC mediating the relationship between WIF and job performance and the relationship between FIW and job performance is not confirmed (−0.014, boot 95% CI= [−0.058, 0.030]; −0.011, boot 95% CI= [−0.054, 0.034]). Therefore, H6a and H6b are not supported.

Given that gender, dependent children and average working hours may affect the mediating effect of AOC, we took gender, dependent children and average working hours as moderating variables to further explore the impact of these three variables on the mediating effect of AOC. Specifically, we also used the PROCESS mediation macro in SPSS to implement a mediating analysis which contains the moderating variables [92]. The analysis results are shown in Table 7. The bootstrap sample was also set to 5000 and the statistical significance was evaluated using a 95% confidence interval (CI) [93,94]. As can be seen from Table 7, the moderating effect of gender is not significant (WIF×gender→job satisfaction, −0.052, *p* > 0.05; FIW×gender→job satisfaction, −0.042, *p* > 0.05; WIF×gender→job performance, −0.067, *p* > 0.05; FIW×gender→job performance, −0.036, *p* > 0.05). The moderating effect of dependent children is not significant (WIF×dependent children→job satisfaction, 0.027, *p* > 0.05; FIW×dependent children→job satisfaction, 0.071, *p* > 0.05; WIF×dependent children→job performance, −0.037, *p* > 0.05; FIW×dependent children→job performance, −0.020, *p* > 0.05).

The moderating effect of average working hours on the relationship between WFC and job performance is not significant (WIF×average working hours→job performance, −0.074, *p* > 0.05; FIW×average working hours→job performance, −0.109, *p* > 0.05), while the moderating effect of average working hours on the relationship between WFC and job satisfaction is significant (WIF×average working hours→job satisfaction, 0.217, *p* = 0.000; FIW×average working hours→job satisfaction, 0.168, *p* < 0.01). Therefore, the average working time can positively moderate the negative relationship between WFC and job satisfaction. This means that the longer the average working time, the stronger the negative relationship between WFC and job satisfaction.

## 6. Discussion

### 6.1. Effects of WFC on Job Outcomes and AOC

The results show that both WIF and FIW have negative effects on AOC and job satisfaction but no significant effects on job performance. These findings are interesting and expand our understanding of the effects of WFC in the construction industry, because previous research on WFC generally believed that it was negatively correlated with AOC, job satisfaction, and job performance [16,55,58,75]. Construction industry is a labor-intensive and task-driven industry, characterized by high risks, heavy workloads, and long construction periods. Compared with other industries, the demanding working environment in the construction industry involves longer average working hours [2,3]. This is because construction projects have the temporary, uncertain and dynamic nature and the tendency to be large-scale, complex and integrated [4]. These characteristics of construction projects lead to difficult tasks, complex processes and unforeseen problems during the projects implementation process. In order to achieve the smooth delivery of the project, many construction enterprises encourage construction professionals to devote more energy and time at work. Therefore, construction professionals work much longer hours than contracted, which limits their abilities to effectively fulfill their family responsibilities. Meanwhile, construction professionals are faced with demands from the family domain, such as raising children, accompanying spouses, and caring for elderly family members.

Such family demands are likely to affect their work. Therefore, it is inevitable for construction professionals to experience WIF and FIW. In the context of Chinese construction projects, the insignificant effects of FIW and WIF on job performance are related to Chinese cultural factors and national conditions. In Western culture, the responsibilities of work and family tend to be separated [8], whereas they are closely linked in Chinese culture [96], which generally believes that success in a career is more important than in personal life. Chinese culture also emphasizes personal devotion and sacrifices family life for work. Moreover, it is generally believed that work contributes to family rather than competing with family life in China [8]. The underlying explanation is that a career is more important than family life to an individual, and having a good career brings good financial support to the family and improves the quality of family life. In this case, having a good career is an expectation held by the family [17]. Therefore, in Chinese culture, the main purpose of an individual is to make a living instead of enjoying life. Additionally, China is still a developing country despite having experienced decades of economic reform, so work remains the primary means of maintaining and improving living standards [5]. For most people, work is the means by which they make a living and support their families, so they must abide by organizational arrangements and undertake tasks as required by their organizations. Therefore, for construction professionals, regardless of their family life’s interference with their work or their work’s interference with their family life, they try their best to complete tasks on time, achieve good work performance, and be responsible for the quality, cost, duration, and safety objectives of projects so as to secure career development and opportunities for promotions.

Because of the adverse consequences of conflicts between the roles, WIF and FIW lead to low levels of AOC and job satisfaction. According to inter-role conflict theory [24], WIF always makes it impossible for construction professionals to effectively perform their family duties, such as accompanying spouses, raising children, and caring for elderly family members [17]. Family stress and negative emotions (e.g., disappointment, frustration, guilt) brought about by WIF will decrease construction professionals’ job satisfaction and the levels of their emotional attachment to their organizations. The negative effects of FIW on job satisfaction and AOC are derived from the interference of family life with work, which negatively affects task completion and ultimately leads to anxiety, irritation, and even, anger. Such negative emotions would have negative effects on job satisfaction and emotional attachment to the organizations.

### 6.2. Effects of AOC on Job Outcomes

The results show that AOC is positively correlated with job satisfaction and job performance. This not only clarifies the relationship of construction professionals’ AOC with job satisfaction and job performance but also fills the gap in the research on the relationship of AOC with job satisfaction and job performance in the context of construction projects. The construction project is a temporary system composed of multi-disciplinary professionals and a project team has the characteristics of diversity, multi-disciplinary knowledge, dynamics, and temporality [17]. The ultimate goal of a project team is to achieve project success by controlling project costs, duration and quality, and improving the economic, social, and environmental benefits of the project [4]. Therefore, for construction professionals, they must face traditional control objectives such as project quality, time and cost, as well as increasingly urgent safety and environmental issues. This brings great pressure to them and makes them unable to fulfill their family responsibilities effectively, which may eventually lead to their job burnout. In this case, AOC of construction professionals becomes very important.

Construction professionals with high levels of AOC tend to have a sense of belonging and altruism, which makes them largely unaffected by the negative effects of heavy pressures at work [97]. Additionally, construction professionals with high levels of emotional attachment to their organizations not only identify with but also show enthusiasm and passion for their organizations and are willing to remain as members [35]. They believe that work includes a wider range of behaviors, such as extra-role behaviors, and tend to participate in more organizational actions and act in the best interests of their organizations [20]. Therefore, construction professionals with high levels of AOC are not only competent and satisfied with their work but also work harder to complete project tasks, improve job performance, and make contributions, such as innovatively completing difficult project tasks, achieving key project nodes ahead of time, and helping save project costs, beyond the normal requirements of their jobs.

### 6.3. The Mediating Role of AOC

The results show that AOC plays a mediating role between WFC and job satisfaction, which reveals the importance of an emotional and psychological connection between employees and organizations. This finding complements the existing body of knowledge on WFC in the field of construction project management by exploring why and how AOC affect the relationship between WFC and job outcomes. As a driving force, AOC is manifested in employees’ desire for organizational membership, willingness to work for their organization, belief in organizational values and goals, and emotional attachment to their organization [70]. Employees with high AOC have been shown to exert more effort on behalf of their organizations in order to achieve the organizational objectives, even when they face difficulties and adversities [98].

In the construction industry, due to the uncertainty, complexity, and high risk of construction projects, construction professionals face many complex tasks and processes, and unforeseen project situations during project implementation [5]. Moreover, construction professionals have important responsibilities for the cost, duration, quality, and safety objectives of a construction project [4]. As a result, construction professionals work long and irregular hours and experience significant stress from work, organization, and society over a long period, from the start of a project to delivery. These factors combine to cause WIF. Meanwhile, many construction professionals are faced with demands from the family domain, such as raising children, accompanying spouses, and caring for elderly family members [9]. Such family demands are likely to affect their work, ultimately leading to FIW. In the context of construction projects, construction professionals with low WFC tend to have a high level of AOC, which makes them have a strong identification with the organization’s goals and values, as well as a high level of job satisfaction [99]. The underlying explanation is that construction professionals tend to have strong AOC when they feel that their work experience, sense of accomplishment, and sense of belonging are in line with their expectations. This can not only enhance the team spirit and organizational cohesion, but also improve their job satisfaction, and ultimately contribute to project success.

## 7. Conclusions and Implications

### 7.1. Conclusions

The present study used SEM to empirically analyze and to discuss the effects of WFC on job satisfaction and job performance and the mediating role of AOC in the effects of WFC on these two job outcomes. The results show that two interrelated dimensions of WFC, namely, WIF and FIW have significant negative effects on AOC and job satisfaction but insignificant effects on job performance. A high level of AOC leads to high job satisfaction and job performance. Furthermore, AOC plays a mediating role between WFC and job satisfaction. This study not only enrich the existing knowledge body of WFC but also contribute to an understanding of how AOC explains the effects of WFC on job outcomes in the context of construction projects, which could help project-oriented organizations better manage their employees’ work–family interfaces and promote their levels of AOC.

### 7.2. Theoretical Implications

This study contributes to the research on WFC, AOC, and job outcome by linking these three concepts in the context of construction projects. First, this study extends the existing body of knowledge on WFC through its investigation of WFC as an antecedent and AOC as a mediating variable between WFC and job outcome. The results verify the dysfunctional role of WFC and advance our understanding of how and why WFC produce dysfunctional effects on employees’ job outcomes in the context of construction projects. In addition, this study demonstrates the importance of considering cultural factors for interpretations of the differences in the consequences of WFC and contributes to a better understanding of the role of culture in research on WFC.

Second, this study explored the mediating role of AOC in the effects of WFC on job satisfaction and job performance of construction professionals. The results reveal the functional role and mediating role of AOC. Because of the dynamic nature, as well as the uncertain internal and external environments, of construction projects, the AOC of construction professionals is critical to project performance, and hence, deserves a closer look. These findings demonstrate the importance of AOC to individual behaviors and attitudes in the context of construction projects. Third, although job outcome has been highlighted as an important topic in the field of construction project, only a limited number of studies have investigated its relationship to individual-related antecedents. This study supplements those about construction professionals’ job outcomes in the context of construction projects.

### 7.3. Practical Implications

The theoretical model developed by and the findings of this study have practical implications for both construction enterprises and construction professionals. First, construction enterprises should pay sufficient attention to issues regarding WFC of construction professionals, strive to become family-friendly organizations, and create family-supported organizational cultures. Considering that construction projects have the characteristics of inflexible scheduling and the inflexibility of the task schedule, some family-friendly measures, such as flexible schedules that have been implemented in some traditional permanent organizations, may seem impractical in the construction industry. However, other alternative work–family balance measures can be designed according to the characteristics of construction projects. One example would be sufficient time given to construction professionals so that they could accompany their families before participating in the next project. Meanwhile, construction enterprises can consider reducing the overtime of construction professionals to ensure that they have sufficient amounts of personal time. If they must work overtime, they should be given appropriate compensation in the form of bonuses, family-related leaves, or promotion opportunities. Additionally, some family-support measures, such as family health welfare, as well as childcare, and eldercare assistance, could reduce the level of WFC.

Second, construction enterprises should also adopt a focus on the functional role of AOC and strengthen the cultivation of AOC for construction professionals to ensure their emotional identification with and attachment to the enterprises, thereby promoting their job performance and job satisfaction. Some ways of strengthening AOC are identifying construction professionals’ career development needs, providing adequate development opportunities, granting full decision-making rights, having open and transparent organizational procedures, and adopting fair incentives. Third, construction enterprises should establish and improve two-way communication mechanisms between them and their employees to better understand their employees’ difficulties and demands in work and life, then provide support and help. For example, formal briefing sessions and value-engineering workshops can be held regularly to review all the project difficulties encountered, as well as discuss solutions and optimization measures, in order to ensure a smooth development of construction professionals’ work. Informal one-on-one interviews between employees and organizations can also be held regularly for discussions of their work and family difficulties so that the organizations can understand the specific difficulties and demands of their work and family, as well as offer help. With such measures, it is expected that the WFC of construction professionals could be reduced and their AOC could be strengthened to prevent the indirect effects of WFC on their job outcomes, thus promoting project success.

### 7.4. Limitations and Future Work

Research on WFC has been conducted for decades but few studies have explored the effect of WFC on job satisfaction and job performance, as well as the possible mediating role of AOC in the effects of WFC on these two job outcomes in the context of construction projects. This study fills this gap by introducing AOC as a mediator variable to develop and verify the theoretical model. Not only has this study validated some existing results in the context of construction projects, but it has also revealed some new and important findings. However, there are still some limitations to this study.

First, the sample data is limited to specific regions in China. It is suggested that the future direction is to collect data from different countries or regions to explore the relationship between WFC, AOC and job outcomes from different cultural perspectives. Second, this study considered only the effects of WFC on job outcome. Independent variables such as work–family facilitation and work–family guilt were not included in the model. Therefore, these variables should be fully considered in future research to determine the effects of work–family interface on job outcomes. Third, employees’ WFC and AOC are evolving and becoming even more complex in some specific situations. Future studies can explore the evolution of the mechanism between WIF and FIW, as well as the driving mechanism of AOC. Fourth, this study did not consider the influence of personality traits, such as the big-five personality traits, on research results. Therefore, future work can incorporate personality traits into study and explore the impact of WFC on job outcomes under the influence of different personality traits. Despite these limitations, the conclusions of this study provide useful references for construction enterprises to help them develop effective strategies for managing WFC and promoting AOC.

## Figures and Tables

**Table 1:** Measurements for WFC, AOC, and job outcomes.

Variables	No.	Measurement	References
Work domain interferes with family domain (WIF)	WIF-1	The demands of work interfere with my family life.	Boles et al. [74]; Netemeyer et al. [60]
	WIF-2	Work takes up much of my time, so it is difficult for me to fulfill my family duties.	
	WIF-3	Because of the demands of my work, what I want to do at home cannot be done.	
	WIF-4	The pressure of work makes it difficult for me to fulfill my family duties.	
	WIF-5	I must change my plans for family activities because of work-related duties.	
Family domain interferes with work domain (FIW)	FIW-1	The demands of my family or spouse/partner interfere with work-related activities.	
	FIW-2	I must put off work because of my family’s demands on my time.	
	FIW-3	Because of the demands of my family or spouse/partner, I cannot accomplish my work on time.	
	FIW-4	My family life interferes with my duties at work, such as arriving at work on time, completing daily tasks, and working overtime.	
	FIW-5	The pressure from my family interferes with my ability to fulfill my job’s duties.	
Affective organizational commitment (AOC)	AOC-1	My values are similar to those of the construction enterprise where I work.	Meyer et al. [18]; Mowday et al. [29]
	AOC-2	I am very concerned about the future of the construction enterprise where I work.	
	AOC-3	I am proud to tell other people that I work for this construction enterprise.	
	AOC-4	Achieving project goals is as important to me as it is to the project.	
	AOC-5	I am willing to work harder than ever to help this construction enterprise make progress.	
	AOC-6	For me, this is the best of all possible construction enterprises for which to work.	
Job satisfaction (JS)	JS-1	Generally speaking, I am satisfied with my present job.	Babin and Boles [75]; Hartline and Ferrell [76]; Leung et al. [30]
	JS-2	I am satisfied with my leader.	
	JS-3	I am satisfied with my work environment.	
	JS-4	I have good relationships with my co-workers.	
	JS-5	I am satisfied with my salary.	
	JS-6	I am satisfied with my chances for promotion.	
Job performance (JP)	JP-1	I am an excellent employee.	
	JP-2	I am in the top ten in terms of employee performance.	
	JP-3	The degree of the completion of my work tasks is very high.	
	JP-4	My cooperation with my team members is very good.	
	JP-5	I know more about the products/services offered to the owner.	
	JP-6	I know the owner’s expectations.	

**Table 2:** Demographic characteristics of respondents.

Characteristic	Category	Frequency	%
Gender	Male	218	68.69
	Female	99	31.31
Age	<30	104	32.81
	30–39	151	47.63
	40–50	39	12.30
	>50	23	7.26
Marital status	Single	68	21.45
	Married	249	78.55
Dependent children (aged 18 years or below)	Yes	221	69.71
	No	96	30.29
Elderly dependents	Yes	214	67.51
	No	103	32.49
Job position	Project engineer	111	35.15
	Department manager	105	33.13
	Project manager	77	24.16
	Others	24	7.56
Average working hours per week	<40 h	17	5.36
	41–50 h	42	13.25
	51–60 h	93	29.34
	>60 h	165	52.05

**Table 3:** Results of confirmatory factor analysis.

Variables	CR	AVE	Fit Indices
WIF	0.86	0.67	$\chi^{2}/df=2.36;$ RMSEA = 0.054; GFI = 0.91; AGFI = 0.90; NFI =0.92; IFI = 0.94; CFI = 0.96
FIW	0.91	0.65	χ^2^*/df* = 2.49; RMSEA = 0.061; GFI = 0.90; AGFI = 0.92; NFI = 0.91; IFI = 0.93; CFI = 0.95
AOC	0.83	0.61	$\chi^{2}/df=1.96;$ RMSEA = 0.051; GFI = 0.94; AGFI = 0.91; NFI =0.93; IFI = 0.95; CFI = 0.91
JS	0.79	0.60	$\chi^{2}/df=2.73;$ RMSEA = 0.076; GFI = 0.92; AGFI = 0.90; NFI =0.91; IFI = 0.93; CFI = 0.90
JP	0.84	0.68	$\chi^{2}/df=1.83;$ RMSEA = 0.049; GFI = 0.91; AGFI = 0.90; NFI = 0.94; IFI = 0.92; CFI = 0.96

Note: CR, construct reliability; AVE, average variance extracted; JS, job satisfaction. JP, job performance.

**Table 4:** The results of independent samples t-test.

Variables	t-test for Equality of Means			
	t	Sig.	Lower 95% CI	Upper 95% CI
WIF	−1.721	0.084	−0.476	0.038
FIW	−1.641	0.078	−0.329	0.027
AOC	−1.493	0.092	−0.462	0.049
JS	−1.719	0.067	−0.314	0.036
JP	−1.540	0.075	−0.410	0.023

Note: JS, job satisfaction. JP, job performance. CI, Confidence interval.

**Table 5:** Results of theoretical model analysis.

Hypothesis	Path Coefficients	C.R. Values	*p* Values	Hypotheses Decision
WIF→JS	−0.139 *	−2.528	0.011	H1a: Supported
FIW→JS	−0.126 **	−3.289	0.001	H1b: Supported
WIF→JP	−0.069	−1.462	0.144	H2a: Not Supported
FIW→JP	−0.070	−1.600	0.110	H2b: Not Supported
WIF→AOC	−0.186 ***	−3.464	0.000	H3a: Supported
FIW→AOC	−0.184 ***	−4.230	0.000	H3b: Supported
AOC→JS	0.416 ***	7.250	0.000	H4a: Supported
AOC→JP	0.353 ***	5.503	0.000	H4b: Supported
FIW→WIF WIF→FIW	0.741 *** 0.835 ***	13.181 13.164	0.000 0.000	—
				—
Fit indices (the full model)	$x^{2}/\mathrm{df}\;=\;1.74$; RMSEA = 0.063; GFI = 0.94; AGFI = 0.92; NFI =0.96; IFI = 0.93			

Note: JS, job satisfaction. JP, job performance. C.R., critical ratio. *, *p* < 0.05. **, *p* < 0.01. ***, *p* < 0.001.

**Table 6:** Results of mediational analysis.

Source	Product of Coefficients		Boot 95% CI		Hypotheses Decision
	Estimate	Boot SE	Lower	Upper	
Mediation: AOC					
Between WIF and job satisfaction					
Indirect effect	−0.114	0.027	−0.170	−0.068	H5a: Supported
Between FIW and job satisfaction					
Indirect effect	−0.115	0.026	−0.172	−0.069	H5b: Supported
Between WIF and job performance					
Indirect effect	−0.014	0.022	−0.058	0.030	H6a: Not Supported
Between FIW and job performance					
Indirect effect	−0.011	0.022	−0.054	0.034	H6b: Not Supported

Note: 5000 bootstrap samples. SE, standard error. CI, confidence interval.

**Table 7:** Results of AOC’s mediational analysis containing the moderating variables.

Source	Path Coefficient	T Statistics	*p* Values
WIF×gender→job satisfaction	−0.052	−1.375	0.126
FIW×gender→job satisfaction	−0.042	−1.170	0.106
WIF×gender→job performance	−0.067	−1.659	0.102
FIW×gender→job performance	−0.036	−1.019	0.137
WIF×AWH→job satisfaction	0.217 ***	7.697	0.000
FIW×AWH→job satisfaction	0.168 **	3.284	0.001
WIF×AWH→job performance	−0.074	−1.485	0.161
FIW×AWH→job performance	−0.109	−1.571	0.142
WIF×DC→job satisfaction	0.027	−1.005	0.116
FIW×DC→job satisfaction	0.071	−1.408	0.270
WIF×DC→job performance	−0.037	−1.029	0.104
FIW×DC→job performance	−0.020	−1.003	0.101

Note: 5000 bootstrap samples. AWH, average working hours. DC, dependent children. **, *p* < 0.01. ***, *p* < 0.001.
